# Supplementary material for: Moral Distress Scores of Nurses Working in Intensive Care Units for Adults Using Corley’s Scale: A Systematic Review
Source: Int J Environ Res Public Health. 2022 Aug 26;19(17):10640. doi: 10.3390/ijerph191710640 (PMC9517876; doi:10.3390/ijerph191710640)
Supplement: Supplementary file 1 [file ijerph-19-10640-s001.zip › ijerph-1845032-supplementary file S1.pdf]

PRISMA 2020 flow diagram for new systematic reviews which included searches of databases, registers and other sources

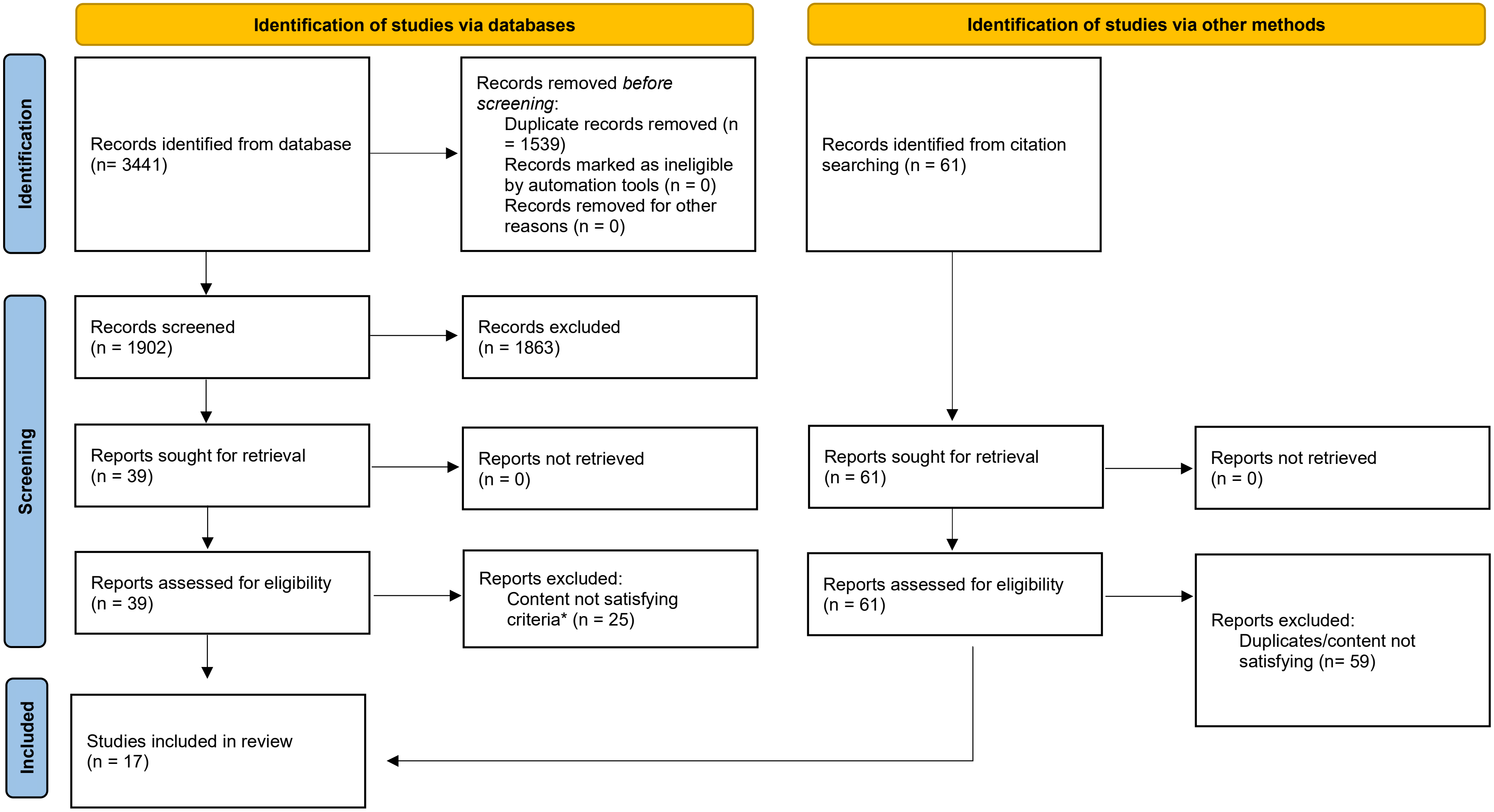

* Articles with validation, cross-cultural adaptation design or correlational design (n=6)
Articles mainly focusing on registered nurses or other healthcare professionals working in hospitals or primary care (not in Intensive Care Units) (n=14)
Articles mainly focusing on moral distress scores, assessed by another instrument (n=4)
Publications not in English or Italian (n=1)

From: Page MJ, McKenzie JE, Bossuyt PM, Boutron I, Hoffmann TC, Mulrow CD, et al. The PRISMA 2020 statement: an updated guideline for reporting systematic reviews. BMJ 2021;372:n71. doi: 10.1136/bmj.n71. For more information, visit: http://www.prisma-statement.org/
