# Supplementary material for: Moral Distress Scores of Nurses Working in Intensive Care Units for Adults Using Corley’s Scale: A Systematic Review
Source: Int J Environ Res Public Health. 2022 Aug 26;19(17):10640. doi: 10.3390/ijerph191710640 (PMC9517876; doi:10.3390/ijerph191710640)
Supplement: Supplementary file 1 [file ijerph-19-10640-s001.zip › ijerph-1845032-supplementary file S2.pdf]

Newcastle-Ottawa Scale adapted for cross-sectional studies

Selection: (Maximum 5 stars)

1) Representativeness of the sample:
    a) Truly representative of the average in the target population. * (all subjects or random sampling)
    b) Somewhat representative of the average in the target population. * (non-random sampling)
    c) Selected group of users.
    d) No description of the sampling strategy.

2) Sample size:
    a) Justified and satisfactory. *
    b) Not justified.

3) Non-respondents:
    a) Comparability between respondents and non-respondents characteristics is established, and the response rate is satisfactory. *
    b) The response rate is unsatisfactory, or the comparability between respondents and non-respondents is unsatisfactory.
    c) No description of the response rate or the characteristics of the responders and the non-responders.

4) Ascertainment of the exposure (risk factor):
    a) Validated measurement tool. **
    b) Non-validated measurement tool, but the tool is available or described.*
    c) No description of the measurement tool.

Comparability: (Maximum 2 stars)

1) The subjects in different outcome groups are comparable, based on the study design or analysis. Confounding factors are controlled.
    a) The study controls for the most important factor (select one). *
    b) The study control for any additional factor. *

Outcome: (Maximum 3 stars)

1) Assessment of the outcome:
    a) Independent blind assessment. **
    b) Record linkage. **
    c) Self report. *
    d) No description.

2) Statistical test:
    a) The statistical test used to analyze the data is clearly described and appropriate, and the measurement of the association is presented, including confidence intervals and the probability level (p value). *
    b) The statistical test is not appropriate, not described or incomplete.

This scale has been adapted from the Newcastle-Ottawa Quality Assessment Scale for cohort studies to perform a quality assessment of cross-sectional studies for the systematic review, “Are Healthcare Workers’ Intentions to Vaccinate Related to their Knowledge, Beliefs and Attitudes? A Systematic Review”.
